# Cancer-associated macrophage-like cells as a prognostic biomarker in solid tumors

**DOI:** 10.1016/j.jlb.2024.100275

**Published:** 2024-11-14

**Authors:** Anthony Pirrello, Murray Killingsworth, Kevin Spring, John E.J. Rasko, Dannel Yeo

**Affiliations:** a Li Ka Shing Cell and Gene Therapy Program, The University of Sydney, Camperdown, 2050, NSW, Australia; b Precision Oncology Laboratory, Centenary Institute, Camperdown, 2050, NSW, Australia; c Department of Anatomical Pathology, NSW Health Pathology, Liverpool, 2170, NSW, Australia; d Medical Oncology Group, Liverpool Clinical School, Western Sydney University and Ingham Institute for Applied Medical Research, Liverpool, 2170, NSW, Australia; e Faculty of Medicine and Health, The University of Sydney, Camperdown, 2050, NSW, Australia; f Cell and Molecular Therapies, Royal Prince Alfred Hospital, Camperdown, 2050, NSW, Australia; g Gene and Stem Cell Therapy Program, Centenary Institute, Camperdown, 2050, NSW, Australia

**Keywords:** Liquid biopsy, Biomarker, Circulating tumor cells, Precision medicine

## Abstract

Cancer-associated macrophage-like cells (CAMLs) are myeloid-lineage cells associated with cancer-derived material that are detectable in the blood. In addition to circulating tumor cells, CAMLs are a promising liquid biopsy biomarker which may assist with prognostication for patient stratification and monitoring response to chemotherapy and radiotherapy in solid tumors. CAMLs have been detected in blood samples from patients with various tumors including lung, pancreas, breast, oesophageal, and colorectal cancers, and to date have not been detected in healthy individuals. However, the optimal method of detection, their origin, function in the circulation, and ultimate utility have not been fully elucidated. This review provides an overview of CAML-related studies and explores their future potential to guide clinical decision-making.

## 1 Introduction

Cancer-associated macrophage-like cells (CAMLs) are giant cells of myeloid lineage found in the blood of cancer patients. CAMLs were first described in 2014 as circulating cells presenting with ‘atypical or multiple nuclei’ and expression of macrophage and tumor markers [1]. Although it is unknown how or when CAMLs arise in the circulation, they are believed to represent tumor-associated macrophages (TAMs) that have phagocytosed tumor cell material locally and have disseminated.

CAMLs may be a useful prognostic biomarker as studies have found an association with poor prognosis, advanced pathological stage, poor progression-free survival (PFS) and overall survival (OS) [[2], [3], [4], [5]]. Furthermore, their presence has been used as a monitoring biomarker where an increase in CAML number and size was found in patients undergoing chemotherapy and radiotherapy [1,2,5,6]. There is currently no gold-standard method for detecting CAMLs and there are limited studies that characterize CAMLs. Herein, this review summarises the current evidence for CAMLs as a cancer biomarker and explores their potential clinical applications.

## 2 What is a cancer-associated macrophage-like cell?

### 2.1 Early studies

The Adams et al. study (2014) defined CAMLs as giant circulating cells of myeloid lineage expressing macrophage- and tumor-specific antigens (CD14/CD11c and CK/EpCAM respectively) [1]. However, prior to this study, cells, similar to CAMLs, have been reported as early as the 1950s. Sandberg et al. first observed cells using Wright-Giesma staining, defining them as either immature haemopoietic cells or epithelial/endothelial cells appearing in peripheral blood samples [7]. Myeloid cells with malignant-like hyperchromatic round and oval nuclei were observed in a breast cancer patient, with morphology resembling those of the primary tumor [8].

Recent immunophenotyping studies of these cells using macrophage markers identified them as macrophage-like cells. For example, Kaposi sarcoma cells stained positive for macrophage antigens: CD68, CD14, PAM-1, and macrophage mannose receptor [9,10]. The expression of additional macrophage antigens, including CD168, MAC387, and DAP12, on breast and rectal cancer cell populations followed [[11], [12], [13]]. Another recent study on blood samples from breast cancer patients used macrophage antigen: CD68, leukocyte antigen: CD45, and epithelial malignancy marker cytokeratin (CK): 8, 18, and 19 on large, granular cells appearing as monocyte-/macrophage-like, which were not observed in normal control samples [14]. Such cells were subsequently demonstrated in mouse models of neovascular vasculogenic mimicry to share similar morphological appearance, phagocytic capability, total lipid composition, and gene expression to macrophages (CD11b, CD68, CD45, CXCR4, Iba1 and F4/80) [15,16].

### 2.2 CAML nomenclature

**Fig. 1:**
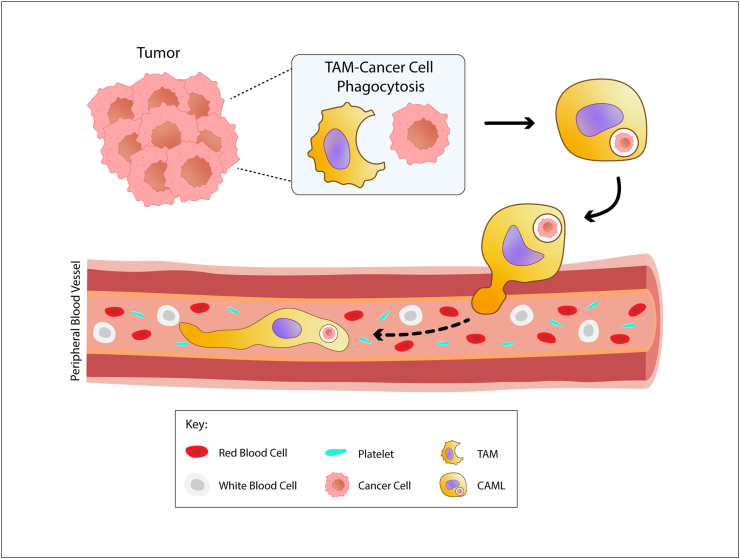
**Cancer-associated macrophage-like cell (CAML) formation following tumor-associated macrophage (TAM)-cancer cell phagocytosis.** CAMLs are thought to be disseminated into the peripheral blood following TAM phagocytosis of cancer cells.

The Adams et al. study from 2014 introduced the term CAMLs [1]. CAMLs can have multiple individual nuclei, fused nucleoli, or a large, atypical nucleus with five morphological cytoplasmic phenotypes: amorphous, oblong, spindle-shaped, round, and tadpole-shaped. CAMLs are hypothesised to be disseminated TAMs (Fig. 1), due to the presence of engulfed organ-specific markers, including PSMA and PDX-1 for prostate and pancreatic cancer respectively. TAMs play a significant role in metastasis, promoting angiogenesis, intravasation, and extravasation, supporting the hypothesis that CAMLs are disseminated TAMs [17]. Cell-cell fusion has also been suggested as an alternative mechanism for CAML formation [18]. Although CAML is the most common term used for these type of cells, other terms have been used including ‘tumacrophage’ [19], ‘multinucleated giant cell’ [20], and ‘circulating stromal cell’ [21,22].

### 2.3 CAMLs compared to other circulating tumor-related cells

Other circulating tumor-related cells, besides CAMLs, have been studied such as circulating tumor cells (CTCs) and circulating hybrid cells (CHCs). Of these, CTCs have been the most extensively analyzed, having been first discovered in 1869 as intact malignant cells disseminated from a tumor [23]. CTCs are believed to be the ‘seeds’ of metastases [[24], [25], [26]]. CTCs have not only been used as a prognostic marker, but also for patient monitoring and treatment selection as part of precision medicine [27].

CHCs are believed to be derived from macrophage heterotypic cellular fusion with tumor cells, retaining characteristics of both cells [28]. Cellular fusion in human cancer was demonstrated in patients following allogeneic bone-marrow transplantation. Alleles from both donor and pre-transplanted lymphocytes were simultaneously demonstrated in tumor cells, indicating formation of a bone marrow-tumor hybrid cell [29,30]. CHCs may be involved in the metastatic cascade, with CHCs shown to navigate from the primary tumor to seed at ectopic sites as metastases in a mouse model [31].

**Fig. 2:**
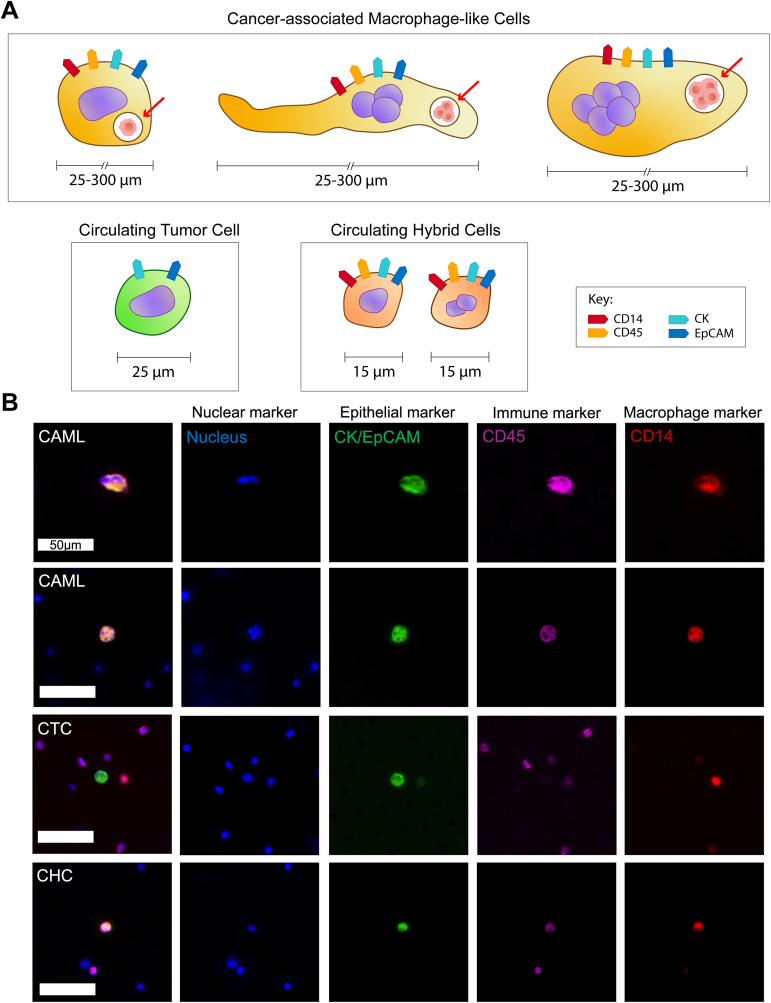
**Schematic and examples of curculating tumor-related cells: cancer-associated macrophage-like cells (CAMLs), circulating tumor cells (CTCs), and circulating hybrid cells (CHCs)**. A: CAMLs can have an atypical single nucleus or multiple nuclei with tumor protein epitopes in cytoplasmic vesicles (red arrow). Typically, smaller CAMLs present as rounded or oval shaped in comparison to their larger counterparts which are more elongated. CAMLs typically range from 25 to 300 μm, marking them significantly larger than CTCs and CHCs. CAMLs and CHCs express the immune surface marker (CD45), macrophage-specific (CD14) and epithelial-specific (cytokeratin (CK) and epithelial cell adhesion molecule (EpCAM)) markers, while CTCs express only epithelial-specific markers. B: Example of CAML, CTC and CHC demonstrating the morphology and marker expressions for each cell type. Peripheral blood samples from pancreatic cancer patients were processed using the CellSieve™ size-exclusion microfiltration method and cells identified using the CyteFinder II microscope with CyteMapper analysis software. Scale bar = 50 μm. (For interpretation of the references to color in this figure legend, the reader is referred to the Web version of this article.)

Unlike CAMLs, both CTCs and CHCs were found to be directly involved in cancer metastasis. Morphologically, CTCs and CHCs are different from CAMLs (Fig. 2). CAMLs are larger in size, ranging from 25 to 300 μm, in comparison to CHCs (5–15 μm) and CTCs (15–25 μm). Both CHCs and CTCs display a typically round morphology whereas CAMLs can be round, oval and with one or two tails [1,31]. Unlike CTCs, which do not express pan-leukocyte marker CD45, CHCs and CAMLs express immune molecules (such as CD45) along with epithelial/tumor markers [31,32]. Specific markers to distinguish between CAMLs and CHCs have not been reported to date. Genotyping may discriminate CHCs from CAMLs, since CHCs are known to lose chromosomes following nuclei fusion, resulting in unique karyotypes for each individual CHC [[33], [34], [35]]. Studies examining all three circulating tumor-related cells concurrently would be beneficial in addressing the differences, advantages and limitations of each of these biomarkers.

## 3 CAML detection and isolation methods

There is no gold-standard CAML isolation and detection method. Methods used for the detection of CTCs have also been used to examine CAMLs. These include size-exclusion, biological-based, or microfluidic techniques to capture CAMLs (Table 1). Such technologies typically include two steps: (1) capture and enrichment, and (2) identification and enumeration. The capture and enrichment step involves either physical interactions between CAMLs and separation mechanisms or antibody-antigen interactions. The identification and enumeration step involves use of immunophenotyping or Romanowsky type stains combined with fluorescence microscopy [1,36]. Microfluidic-based technologies include an additional strategy: (3) capture, release, and characterisation, where isolated CAMLs are used for downstream analysis, such as genomics, transcriptomics, and proteomics. Each of these strategies has limitations. Current methods have detected CAMLs with a range of 0–14 cells/mL of peripheral blood [37], highlighting the challenge for accurate isolation and enrichment. Nonetheless, methods utilized were designed and initially tested for isolation of CTCs. A specifically designed technology is required for the accurate and sensitive detection of CAMLs as well as for their downstream characterization. This will provide further insights into CAML biology for the development of isolation methodologies and evaluation of their clinical utility in precision medicine.

**Table 1:** CAML detection methods and detection rates.

Type of Technology	Detection Technique	Study Year [Reference]	Number of Patients	Total Positive for ≥1 CAML (%)	Mean Detection Rate (%)
**Size-Exclusion-Based Technologies**	CellSieve™	2014 [1]	79	92	85.9
		2016 [37]	41	93	
		2016 [38]	3	100	
		2017 [39]	41	81^a^	
		2020 [5]	32	88	
		2020 [2]	35	91^a^	
		2021 [3]	61	95	
		2022 [40]	30	97	
		2022 [41]	64	98	
		2022 [42]	25	84	
		2022 [43]	53	98	
		2023 [44]	40	100	
		2023 [45]	90	79	
	ScreenCell®	2016 [36]	20	75	54.6
		2020 [46]	40	34.2	
		2022 [47]	9	100	
**Biological-Based Technology**	CellSearch®	2017 [48]	127	16.5	16.5
**Microfluidic-Based Technologies**	Parsortix	2022 [4]	55	40	40
	Labyrinth	2022 [49]	20	85	85
		2023 [50]	21	NR	

a Baseline/pre-treatment detection rate; NR: Not Reported.

### 3.1 Size-exclusion-based technologies

The physical isolation of CAMLs by size-exclusion is the most used technique for detecting CAMLs (Table 1). Such methods capture CAMLs based on differences between cell size, density, and deformability. Size-exclusion technologies, CellSieve™ and ScreenCell®, enrich CAMLs based on low-pressure membrane filtration (pore size: 7 μm and 6.5 μm respectively). CellSieve™ uses an antibody panel consisting of CK, EpCAM, CD45 and CD14, whereas ScreenCell® uses cytomorphological evaluation (May-Grünwald-Giesma stain) [51,52]. Both technologies capture large circulating tumor-associated cells without dependence on surface-marker selection. Living and fixed cells can be isolated by both technologies, enabling genotypical, phenotypical, and functional applications. CellSieve™ had higher mean CAML detection rates (85.8 %) compared to ScreenCell® (54.6 %), where ScreenCell® detection rates had larger variability reported (standard error of the mean: 19.2 for ScreenCell® vs 2.3 for CellSieve™).

Size-exclusion technologies are limited by the potential heterogeneity of CAML size and morphology. CAMLs may potentially squeeze through the filter membrane and elude capture [53]. CAMLs may also clot the filter pores and hinder flow of the sample. Cell morphology may not be maintained during processing, with malformation of cells caused by the pressure-gradient force [54]. Overall, size-exclusion is a technically straightforward methodology which has been utilized in multiple CAML studies with generally high detection rates.

### 3.2 Biological-based technology

Biological-based technologies, such as CellSearch® system, have generally been used for the detection of CTCs. CellSearch® remains one of the most commonly used FDA-approved CTC detection system [55]. CellSearch® relies on antibody-antigen interaction for positive selection, capturing epithelial cell adhesion molecule (EpCAM) expressing cells by immunomagnetic separation. Following enrichment, identification typically occurs by subsequent cytokeratin (CK), CD45 and DAPI staining of cells [56]. Only one study to date has reported CellSearch® for CAML isolation [48], wherein 16.5 % (21/127) of metastatic breast cancer patients were found with detectable CAMLs (Table 1). As detection depends on EpCAM expression, poor detection of low EpCAM-expressing CAMLs may hinder their detection, such as tumor cells that do not express EpCAM or are undergoing epithelial-mesenchymal transition [57].

### 3.3 Microfluidic-based technologies

Microfluidic-based methods have emerged for the label-free isolation of CTCs as well as CAMLs. Such methods apply intrinsic forces for CAML enrichment, rather than extrinsic, followed by identification through different physical and biological properties. The Parsortix™ system is a FDA-approved microfluidic-based technology for the detection of CTCs [27,58]. Circulating tumour cells as well as larger cells, including CAMLs, are caught in the filtration cassette due to size and lower compressibility than leukocytes [59]. Staining with selected antibodies and imaging is possible without removal of cells from the filtration cassette [59]. Captured cells may also be eluted from the cassette for further characterisation including fluorescence in situ hybridization (FISH), quantitative polymerase chain reaction (qPCR), and cell culture. Only one study has utilized Parsortix™ for CAML detection, reporting a 40 % detection rate in pancreatic cancer patients at baseline and 48 % after follow-up [4]. Another microfluidic-based technology, the Labyrinth microfluidic chip, utilizes inertial forces to concentrate CAMLs, CTCs and white blood cells into separate streamlines [60]. Further characterisation of eluted cells is similarly possible with Labyrinth. Two studies have utilized Labyrinth for CAML detection, with only one study reporting yield and purity (Table 1). Nonetheless, both technologies are limited by the high cost, potential of larger cells disrupting flow, and technically challenging processing [59,60].

## 4 CAML clinical studies

### 4.1 Prognosis

The prognostic value of CAMLs has been examined in various solid tumors and disease stages (Table 2) [[1], [2], [3], [4], [5], [6],^36^,^37^,^48^,[40], [41], [42], [43], [44], [46], [47], [49], [50]]. In metastatic breast cancer, detection of CAMLs at baseline was associated with shorter PFS (HR = 1.75, *p* *= 0.0374*) and OS (HR = 3.75, *p* = 0.0042) [48]. A further study in resectable pancreatic cancer patients found that CAML detection only at follow-up, collected during chemotherapy treatment, had worse RFS (HR = 4.3, *p* = 0.023) [4]. Beyond detecting CAMLs, high CAML number, larger size, expression of PD-L1, CXCR4 and CCR5 on CAMLs, presence of micronuclei, and CAMLs bound to CTCs have been found to be associated with worse prognosis (Table 2).

**Table 2:** Prognostic value of CAMLs.

CAML characteristic		Study Year [Reference]	Cancer (Stage)	Number of Patients	Prognostic Value	Study Findings
**CAML Presence**		2017 [48]	Breast (IV)	127	PFS, OS	Presence of CAMLs at baseline associated with shorter PFS (HR = 1.75, *p* = *0.0374*) and OS (HR = 3.75, *p = 0.0042*)
		2022 [4]	Pancreas (I-IV)	55	RFS	Presence of CAMLs at follow-up associated with shorter RFS (HR = 4.3, *p* = 0.023)
		2021 [3]	Pancreas (I-IV)	63	PFS	CAMLs (>12) had worse PFS (HR = 6.09, *p* = 0.002)
**CAML Size (giant CAMLs: ≥50 μm; small CAMLs: < 50 μm)**		2020 [2]	Lung (II-III)	39	PFS, OS, RFS	Giant CAMLs at first follow-up correlated with development of distant metastases (HR = 4.9, *p* = 0.015), RFS (HR = 2.4, p = 0.036), PFS (HR = 2.5, p = 0.025) and OS (HR = 3.5, p = 0.034)
		2020 [5]	Oesophageal (I-III)	32	PFS, OS	Small CAMLs at completion of therapy was linked to improved PFS (HR = 12.0, *p* = 0.004) and OS (HR = 9.0, *p* = 0.019)
		2021 [3]	Pancreas (I-IV)	63	PFS, OS	Giant CAMLs had worse PFS (HR = 3.90, *p* < 0.001) and OS (HR = 2.53, *p* = 0.019)
		2023 [44]	Renal (III-IV)	40	PFS, OS	Giant CAMLs (>70 μm) had worse PFS (HR = 2.84, *p* = 0.027) and OS (HR = 3.95, *p* = 0.015)
		2023 [45]	Prostate (I-IV)	90	PFS, OS	Giant CAMLs had worse PFS (HR = 2.4, *p* = 0.031) and OS (HR = 5.4, *p* < 0.001)
**Prognostic Marker Expression on CAMLs**	**PD-L1 on CAMLs**	2017 [39]	Lung (I-IV)	41	–	PD-L1 expression after radiotherapy not associated with PFS (HR = 0.7, *p* = 0.581)
	**RAD50**	2017 [39]	Lung (I-IV)	41	–	Median PFS was longer in patients after radiotherapy with >1 RAD50 foci/cell (9.8 months vs. 18.5 months). PFS not significant (HR = 2.3; *p* = 0.27)
	**CXCR4**	2022 [40]	Pancreas (I-IV)	30	PFS, OS	High expression of CXCR4 correlated with presence of increased CAMLs (*p* = 0.025), worse PFS (HR = 4.0, *p* = 0.012) and OS (HR = 4.8, *p* = 0.006)
	**≥10 CCR5 Pools**	2022 [43]	Breast (IV)	54	PFS, OS	CCR5 correlated with increased CAML number (*p* = 0.019)
**CAML Micronuclei**		2022 [42]	Colorectal (IV)	25	PFS, OS	Micronuclei correlated with tumor response; PFS (HR = 17.2, *p* = 0.0014) and OS (HR = 70.3, *p* = 0.0027)
**CAMLs Bound CTCs**		2014 [1]	Not specified (I-IV)	26	OS	6 patients with CAML-CTC had worse OS (median = 7 months) compared to those with no CAML-CTC (median at 2 years not reached)

OS: Overall Survival; RFS: Relapse-Free Survival; PFS: Progression-Free Survival; DFS: Disease-Free Survival.

#### 4.1.1 CAMLs compared to other blood biomarkers

CAMLs have been reported to have superior prognostication compared to CTCs when examined in parallel. Mu et al. found that the presence of CAMLs provided better independent prognosis estimates over CTCs in breast cancer patients [48]. Another study in breast cancer found mean CAML counts were higher compared to CTCs; however there was no relationship between CAML and CTC counts as some patients had CAMLs but no CTCs and vice versa [49]. In prostate cancer, CAMLs were detected in more participants (79 %) compared to CTCs (21 %) [45]. In pancreatic cancer, CAMLs were detected in a higher proportion of patients (95 %) compared to CTCs (23 %) as well as standard pancreatic cancer blood biomarkers: CA19-9 (42 %) and CEA (16 %) [3]. The study found that patients with disease progression (75 %) exhibited an increased CAML number at follow-up compared to their baseline levels, whilst patients with stable disease had decreased. Such correlations were not found in the other blood biomarkers. In a follow-up study, CAMLs were detected in 100 % of pancreatic cancer patients with metastatic disease compared to 42 % for CTCs [40]. Another study found a two-fold increase the presence of CAMLs in relapsed patients compared to CA19-9 [4]. Although these represent limited studies, CAMLs may offer improved prognostic estimates compared to other blood-based biomarkers if larger validation studies are undertaken. Combining CAML information with other blood multianalyte biomarkers such as CTCs and ctDNA would be interesting to identify if improvement in the utility of CAMLs could be developed.

#### 4.1.2 Monitoring therapeutic response

CAMLs may also act as a surrogate biomarker to monitor therapeutic response. Higher CAML numbers were found in cancer patients undergoing chemotherapy (10–29 cells/7.5 mL) compared to hormone therapy or untreated patients (3–5 cells/7.5 mL) [1,37]. An increase in CAMLs was also found following chemotherapy in curative and palliative pancreatic cancer patients (9.4 % and 7.1 % respectively) [4]. This is not limited to chemotherapy, with the number of patients with CAMLs increased directly following radiotherapy [39]. Chemotherapy and radiotherapy are known to produce additional tumour cell debris (apoptotic, necrotic, and/or fragmented cells [61,62]) which may result in TAM phagocytosis [63,64]. Adams et al. postulated CAML formation resulted from phagocytosis of cellular debris at the tumour site where CAMLs were found to be increased after chemotherapy and radiotherapy treatment [1].

A difference in CAML size after chemotherapy has also been reported. In unresectable lung cancer patients, the presence of giant CAMLs (≥50 μm) at the time of first follow-up correlated with development of metastases and improved prediction of PFS, OS, distant-failure survival, relapse-free survival in comparison to age, radiation dose, smoking status, and tumor histology [2]. Although analysis was performed at first follow-up, giant CAMLs were observed throughout treatment. In contrast, those with small CAMLs (<50 μm) were less likely to develop metastatic disease. A study in oesophageal cancer found CAML size initially increased after chemoradiotherapy but decreased after treatment completion with giant CAMLs (≥50 μm) found to be an independent prognosis for worse OS after completion of therapy (*p* = 0.0407) [5]. A study in renal cell carcinoma found that a cutoff of 70 μm was most optimal in predicting prognosis where increase in CAML size (from <70 μm to ≥ 70 μm) was associated with PFS (HR = 5.8, *p* = 0.022) [44]. Additional studies with longer follow-up, beyond 2 years, are required to better understand how CAML number, CAML size cutoff, change in CAML size, and markers on CAMLs can monitor therapuetic response as well as to determine the most clinically relevant blood collection timepoint for prognostic utility.

### 4.2 CAMLs for cancer screening

CAML detection as a screening tool could facilitate early cancer detection and improve patient outcomes. Adams et al. found high sensitivity (93 %) and specificity (100 %) when using CAMLs as a screening tool in cancer patients (N = 41) compared to healthy individuals (N = 16) [37]. Another study found a diagnostic accuracy of 96 % (area under the curve (AUC) of receiver operating characteristic (ROC) curve) in cancer patients (N = 63) compared to healthy individuals (N = 40) [3]. So far, studies have not found CAMLs in healthy individuals (combined N = 114) [1,3,37,39,65]. However, a study did report CAMLs in benign breast conditions (N = 5/19), resulting in a lower sensitivity (88 %) and specificity (74 %) for breast cancer detection [37]. As such, this may limit the use of CAMLs as a screening biomarker for solid tumors. Since CAML detection in healthy individuals is well-established, it would be useful for future studies to include those with benign disease to establish whether CAMLs could be used as a screening tool and able to differentiate between benign and malignant conditions.

## 5 Characterization of CAMLs

Characterizing CAMLs beyond immunophenotyping and cell counts is critical to understand their role. Only one study has characterised CAMLs by examining telomere structure in melanoma patients [47]. Two CAML sub-groups were described: one with a very low number of short telomeres, similar to leukocytes, and the other with abnormally high levels of genetic instability. As shown in previous studies [[66], [67], [68]], genetic instability may be linked to the presence of DNA from cell fusion or engulfment of apoptotic bodies. This therefore suggests that a proportion of CAMLs are likely to be derived from cell fusions or phagocytic events with apoptotic material. CAMLs were also found to have a higher telomeric distribution compared to CTCs and leukocytes. Telomere structure may therefore be useful in discriminating CAMLs from normal immune cells or other circulating tumor-related cells.

Downstream characterization has been undertaken in CTCs, drastically improving our understanding of how metastases arise. Technological advances have facilitated the genomic and transcriptomic characterization of CTCs at the single cell level [69], however these are yet to be applied to CAMLs. Furthermore, studying the proteome [70] and lipidome [71] at the single cell level is also now possible. Applying these novel single-cell technologies could provide critical insights into the origins of CAMLs, their formation, role and purpose.

### 5.1 CAML microscopic characterization

Characterization of CAMLs by microscopy and immunocytochemistry may provide insights into the lineage of these cells. Of the myriad of microscopy modalities now available, recent attempts to combine information from several modalities have shown synergistic benefits. For example, correlative light and electron microscopy (CLEM) methods may leverage fluorescence microscopy for the detection of nanoparticle-labelled biomarkers with ultrastructural context provided by electron microscopy of the same section or cell surface [72]. Such methods when applied to sections through the cell interior (cytoplasm) will be particularly relevant to the study of CAMLs if these multinucleated single cells are multinucleated giant cells (MGCs) of chronic inflammation [73] or clusters of single nucleated cells with intact cell plasma membranes and intercellular junctions. Information of this type would be essential in revealing the site of origin of these cells.

CAMLs can be further examined ultrastructurally using transmission electron microscopy (TEM) or high-resolution scanning electron microscopy (HRSEM). Ultrastructural resolution may reveal the presence or absence of a plasma membrane between individual nuclei within the CAML and 3D analysis could resolve any multi-lobulation of the nuclei. HRSEM has a large imaging area (5 × 5mm) without the interference of grid bars which occurs with conventional TEM. Furthermore, immunocytochemical staining can be incorporated into the workflow [74] to reveal the ultrastructural localisation of key proteins for identification of cell and organelle phenotype.

## 6 Conclusion

From the early observations of atypical circulating cells to the adoption of the term ‘CAMLs’, investigating the detection and possible clinical value of these circulating cells has been an active area of research over the last decade. The detection of CAMLs has been found to be prognostic in both localised and metastatic solid tumor patients, particularly the presence of giant CAMLs (>50 μm) during or after therapy. Nevertheless, current studies are all retrospective and prospective studies are required to demonstrate their prognostic potential to guide patient management and facilitate early initiation of other therapies for patients likely to progress or unlikely to respond to their current therapy. A gold-standard CAML-specific detection assay is needed through well-designed comparisons of competing technologies. Downstream characterization, including single-cell and advanced microscopy techniques, will enable better understanding of the origin, formation and function of CAMLs. Once validated, CAMLs, as an alternative circulating tumor-related cell biomarker, could enable personalized cancer therapy and guide clinical decision-making to improve cancer patient outcomes.

## Ethics approval

Study involving human participants were reviewed and approved by Sydney Local Health District, Australia (X19-0490). The participants provided their written informed consent to participate in the study.

## Consent for publication

All authors have read and agreed to the published version of the manuscript.

## Availability of data and materials

Data sharing is not applicable to this article. No new data were created or analyzed in this study.

## Code availability

Not applicable.

## Authors’ contributions

All authors were involved in the conceptualisation, writing of the original draft, and reviewing and editing the manuscript.

## Declaration of competing interest

The authors declare the following financial interests/personal relationships which may be considered as potential competing interests: Dannel Yeo reports financial support was provided by Tour de Cure Ltd (RSP-320). Dannel Yeo reports financial support was provided by Sydney Cancer Partners via Cancer Institute NSW (2021/CBG0002). John EJ Rasko reports financial support was provided by Li Ka Shing Foundation. John EJ Rasko reports financial support was provided by National Health and Medical Research Council (Investigator grant: 1177305). John EJ Rasko, Kevin Spring reports financial support was provided by Cancer Council New South Wales (RG20-07). John EJ Rasko reports a relationship with Office of the Gene Technology Regulator that includes: consulting or advisory. John EJ Rasko reports a relationship with Spark Therapeutics Inc that includes: consulting or advisory. John EJ Rasko reports a relationship with Cynata that includes: consulting or advisory. John EJ Rasko reports a relationship with Pfizer Inc that includes: consulting or advisory. John EJ Rasko reports a relationship with RareCyte that includes: equity or stocks. If there are other authors, they declare that they have no known competing financial interests or personal relationships that could have appeared to influence the work reported in this paper.

## Abbreviation List

CAML: Cancer-associated macrophage-like cell
CHC: Circulating hybrid cell
CK: Cytokeratin
CTC: Circulating tumor cell
DFS: Disease-free survival
EpCAM: Epithelial cell adhesion molecule
HREM: High-resolution scanning electron microscopy
OS: Overall Survival
PFS: Progression-free survival
RFS: Relapse-free survival
TAM: Tumor-associated macrophage
TEM: Transmission electron microscopy
